# A Bio-Inspired Two-Layer Sensing Structure of Polypeptide and Multiple-Walled Carbon Nanotube to Sense Small Molecular Gases

**DOI:** 10.3390/s150305390

**Published:** 2015-03-05

**Authors:** Li-Chun Wang, Tseng-Hsiung Su, Cheng-Long Ho, Shang-Ren Yang, Shih-Wen Chiu, Han-Wen Kuo, Kea-Tiong Tang

**Affiliations:** 1 Analytical Chemistry Section, Chung-Shan Institute of Science & Technology, Hsinchu 30325, Taiwan; E-Mails: rubsico@ms28.hinet.net (L.-C.W.); pandasu0926@gmail.com (T.-H.S.); johnnie.ho1213@msa.hinet.net (C.-L.H.); sunmanup@gmail.com (S.-R.Y.); 2 Department of Electrical Engineering, National Tsing Hua University, No. 101, Sec. 2, Kuang-Fu Road, Hsinchu 30013, Taiwan; E-Mail: swchiu1984@gmail.com (S.-W.C.); 3 Department of Chemistry, National Central University, Taoyuan 32001, Taiwan; E-Mail: herman.h.kuo@gmail.com

**Keywords:** bio-inspired, two-layer structure, multiple-walled carbon nanotube (MWCNT), polypeptide, gas sensing

## Abstract

In this paper, we propose a bio-inspired, two-layer, multiple-walled carbon nanotube (MWCNT)-polypeptide composite sensing device. The MWCNT serves as a responsive and conductive layer, and the nonselective polypeptide (40 mer) coating the top of the MWCNT acts as a filter into which small molecular gases pass. Instead of using selective peptides to sense specific odorants, we propose using nonselective, peptide-based sensors to monitor various types of volatile organic compounds. In this study, depending on gas interaction and molecular sizes, the randomly selected polypeptide enabled the recognition of certain polar volatile chemical vapors, such as amines, and the improved discernment of low-concentration gases. The results of our investigation demonstrated that the polypeptide-coated sensors can detect ammonia at a level of several hundred ppm and barely responded to triethylamine.

## 1. Introduction 

In recent years, electronic nose system developers [1,2,3,4,5] have striven to reduce the price and size and increase the reproduction levels and reaction rates of the systems, and to construct systems that simultaneously monitor multiple gases [6,7]. The sensor materials used to construct chemical resistors are divided into two major types: inorganic semiconductors [8,9] and organic polymers [10,11,12], both of which respond to the adsorptive analytes that trigger physical reactions, subsequently changing resistivity or the dielectric constant. These sensing materials can be deposited on a thin film to fabricate chemical resistors. By measuring the resistance changes, the concentration of analytes can be determined. The thin films of functional polymers possess appealing features, such as simple manipulation, high selectivity at room temperature, rapid absorption and desorption of tested gases, and the ability to couple readily with microstructure sensors [13,14,15,16]. To attain a high performance level of sensitivity and selectivity, researchers are developing sensing materials in recognizing volatile organic compounds (VOCs). To achieve this goal, a variety of materials featuring distinct characteristics have been investigated. For example, polymers [17,18] and molecularly imprinted polymers [19] have been used to identify various chemical vapors in several studies.

Using multiple-walled carbon nanotubes (MWCNTs) to detect chemical gases and vapors has been an active area of research [20,21,22,23]. Several devices have been designed to detect resistance changes in the Schottky barriers among nanotubes and metal contacts [24,25]. These devices could be used to improve real-time sensing for monitoring combustible gases, gas leakages, and environmental pollution.

Implementing proteins and polypeptides in biosensors has increased the sensitivity and selectivity of sensors toward certain targets. In recent studies, a peptide, which is specific to the anthrax protective antigen, has been utilized to test the sensitivity and specificity of the biosensor toward protein markers when immobilized on a MWCNT [26]. Peptides can also be used to recognize and measure gases. A previous study reported that several mammalian species, including humans, can recognize gas mixtures comprising thousands of odorants produced by distinct compounds at low concentrations [27]. The mammalian olfactory system, which is one of the most effective gas sensing systems, uses the receptors attached to the cilia of neurons in detecting various gaseous molecules [28]. By studying and understanding the benefits of an olfactory system that can identify and is sensitive to gas odorants, innovative methods have been developed that use this natural design in the manufacturing of sensing devices that monitor low traces of specific chemical vapors. For example, peptides mimicking the binding site of a specific olfactory receptor that is sensitive to a certain gas, such as hexanol, were synthesized [29]. Exploiting these biomimetic-specific peptides requires applying comprehensive research findings on olfactory receptors and the corresponding ligands. However, separating the receptors and ligands individually for study is impractical because of the large amount of olfactory receptors and possible targeted odorants.

To resolve this problem, we propose a peptide-based, nonselective sensing approach. To perform a primitive study, we used a readily available polypeptide, DAEFRHDSGYEVHHQKLVFFAEDVGSNKGAIIGLMVGGVV. Two volatile organic compounds of amines were also tested. The results indicated that the polypeptide-MWCNT film recognized ammonia and distinguished the trimethylamine (TEA) vapor from the gases.

## 2. Experiment 

### 2.1. MWCNT-Polypeptide Gas Sensor 

**Figure 1:**
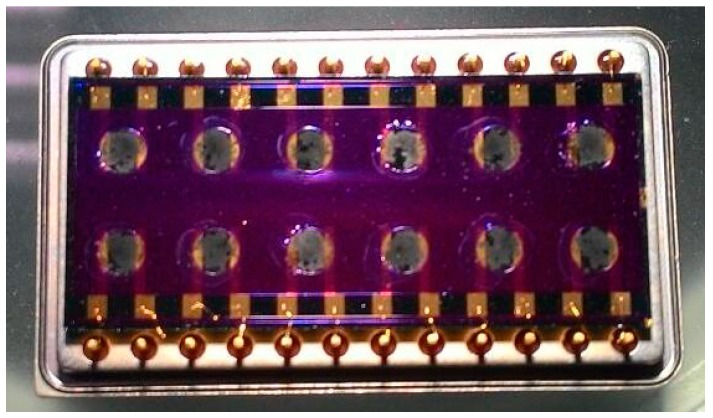
Sensor array chip.

The sensor array chips (Figure 1) were fabricated on a 4-in Si wafer by using a batch process; each sensor chip was 34 mm × 20 mm in area. One gas sensor array chip has 12 independent sensing areas [30,31]. Each circular membrane sensor was limited to 2 mm in diameter to minimize heat loss from the silicon substrate.

We adopted a two-layer film-making method [32]: the MWCNT-modified electrode layer was prepared by drop-casting a methyl ethyl ketone (MEK) solution with 0.1 µL (1 wt%) MWCNT (XinNano Materials, Inc.; approximately 8 nm in diameter, 13 µm long, and >90% pure) onto the desired part of an interdigitated microelectrode on a chip by using a high-performance liquid chromatography syringe. The MEK solvent was evaporated in air at room temperature for 2 h to yield the MWCNT film. The 0.1-µL deionized water solution of polypeptide was then drop-casted onto the MWCNT layer, and the sensor resistance after each casting step was monitored to control the value within a 1 kΩ–100 kΩ range. Finally, the sensor was dried in a vacuum for 24 h to remove the remaining solvent and form a gas sensor.

### 2.2. Experimental Setup 

**Figure 2:**
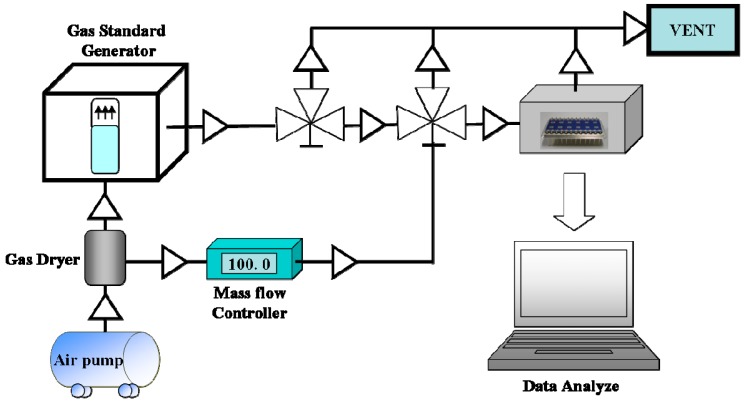
Experimental setup used to characterize the sensing volatile organic compound.

Figure 2 shows the experimental setup used to characterize the VOC-sensing properties of the sensor devices. The test bench comprised the gas delivery system, sensor test chamber, and the computer-controlled electrometer for sensor response acquisition. The gas delivery system included a refrigerating air dryer, pressure regulators, and mass-flow controllers to control the flow rate and concentration of test gas. To conduct the gas sensing experiments, a test stand with a sensor array chip was placed in a glass vessel. The NH_3_ gas was prepared using a certified NH_3_ cylinder (283 ppmv) (San Fu Chemical Co., N_2_ balance) and air. The TEA gas was prepared using a standard gas generator that contained a certified permeation tube with known concentrations generated using a standard gas generator (KIN-TEK Laboratories, Inc. La Marque, TX, USA). The gas concentration was calibrated by measuring the weight loss from the organic solvent solution, and the gas flow rate was controlled using a calibrated mass-flow controller (Aalborg, Inc. Orangeburg, SC, USA) with air as the carrier gas. We conducted the experiments with pure ammonia and TEA gases separately by changing the gas line. For the gas test, after the system was stabilized in dry air, the target gas at a flow rate of 100 mL/min was infused into the chamber (adsorption), followed by air (desorption). After each test run, the chamber was purged with air. All tests were performed in a 3.4 cm^3^ chamber with a controlled temperature (27 °C) and humidity (45% relative humidity). Electrical resistance outputs (△R/R %) from each sensor element were measured using a National Instrument data acquisition card (Interface card: NI DAQ 6009) with a self-written LabVIEW program [32,33].

## 3. Results and Discussion 

In this study, we developed a conductive MWCNT-polypeptide composite sensing material using a two-step approach. The idea of exploiting polypeptides as the outer membrane for gas sensing was inspired by the olfactory organ structure. The mucus layer produced by the mucous cells in the nose comprises various substances that play different roles in the olfactory system. The secreted mucous membrane protects the body by creating a barrier and preventing the inside of the body from losing moisture. Furthermore, the mucus membrane facilitates gas exchange and absorption. Working from this concept of adopting MWCNT as a responsive and conductive layer and polypeptide as a specific filter, we fabricated a polypeptide-based sensor.

### 3.1. Performance of Three MWCNT-Assisted Sensing Membranes

**Figure 3:**
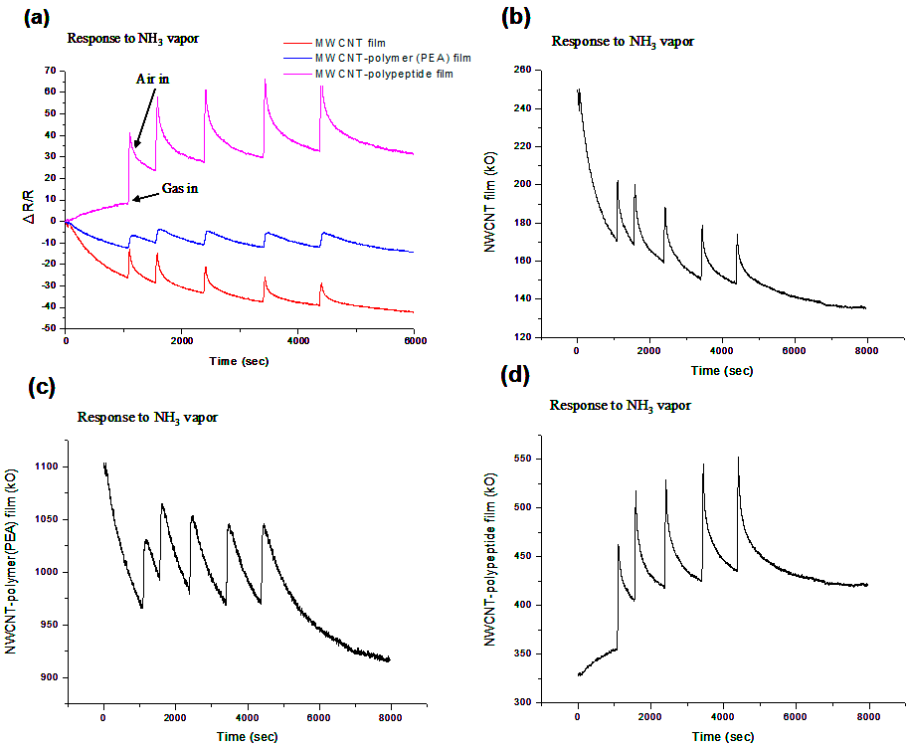
Gas sensing responses to NH_3_ gas (283 ppmv) of (**a**) MWCNT films, MWCNT-poly(ethyl acrylate) (PEA) composite films, and MWCNT-polypeptide composite films; (**b**) MWCNT films; (**c**) MWCNT-PEA composite films; and (**d**) MWCNT-polypeptide composite films.

Figure 3 shows the NH_3_ vapor (283 ppmv) test results of the MWCNT films, MWCNT-poly(ethyl acrylate) (PEA) composite films, and MWCNT-polypeptide composite films, the gas response percentage curves of which were approximately 8%, 5%, and 25%, respectively. The sensing behavior of the MWCNT films and MWCNT-PEA composite films was similar to that previously reported [34,35,36]. The baseline value of the MWCNT films and MWCNT-PEA composite films varied with time and recovered slowly after NH_3_ gas injection. Compared to the other two sensing materials, the MWCNT-polypeptide composite films exhibited higher reactivity, sensitizing, and desensitizing behavior.

### 3.2. Effect of Polypeptide Ratios on Performance of MWNCT-Polypeptide Sensors

**Figure 4:**
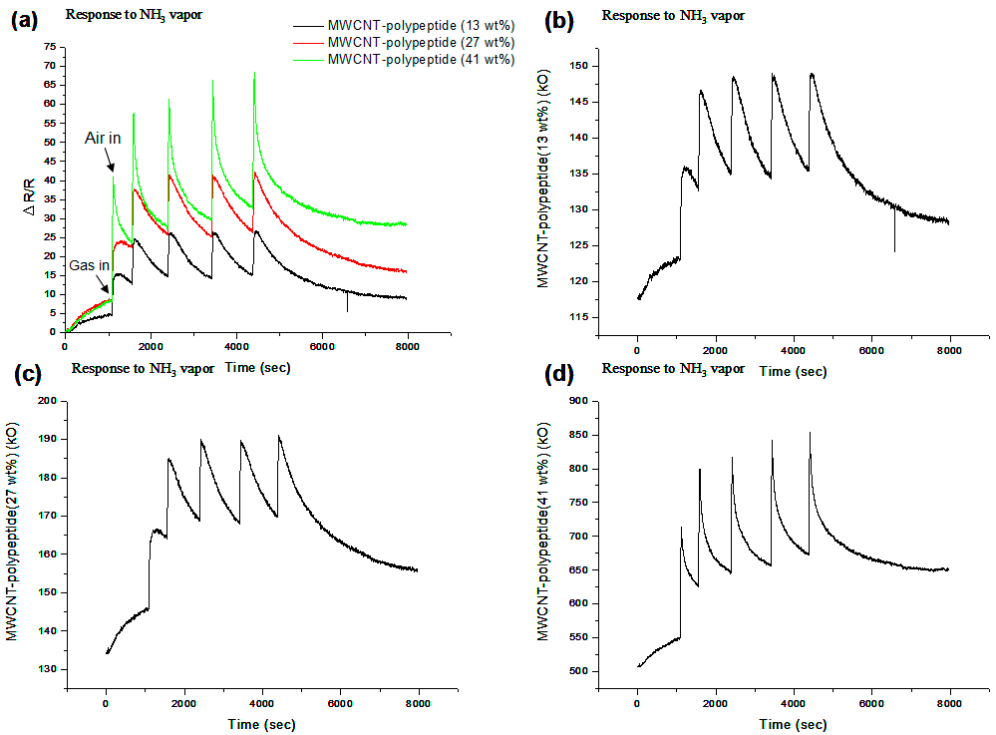
Gas sensing responses to NH_3_ gas (283 ppm) of (**a**) MWCNT-polypeptide composite films with polypeptide concentrations of 13.0 wt%, 27.0 wt%, and 41.0 wt%; (**b**) MWCNT-polypeptide (13.0 wt%) composite films; (**c**) MWCNT-polypeptide (27.0 wt%) composite films; and (**d**) MWCNT-polypeptide (41.0 wt%) composite films.

**Figure 5:**
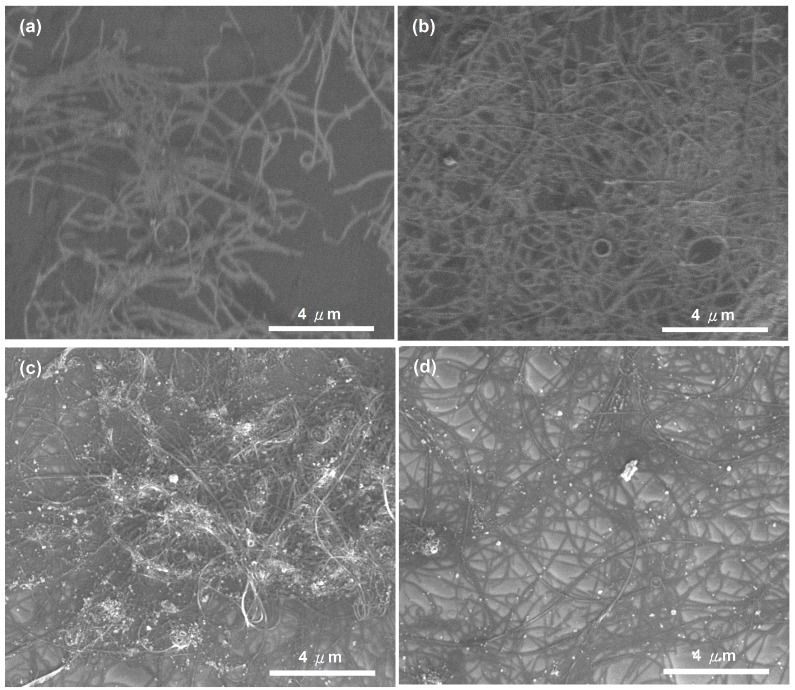
Scanning electron microscope-captured surface morphologies of (**a**) MWCNT; (**b**) MWCNT-polypeptide (13.0 wt%) composite films; (**c**) MWCNT-polypeptide (27.0 wt%) composite films; and (**d**) MWCNT-polypeptide (41.0 wt%) composite films.

We compared the performances of the MWNCT-polypeptide sensors, prepared by drop-casting 1 wt% MWCNT solution as the base layer and various wt% polypeptide (13.0 wt%, 27.0 wt%, and 41.0 wt%) solutions as the top layer. Figure 4 shows the NH_3_ gas test results of the three membranes. After introducing the NH_3_ gas, sensor resistances increased gradually, peaking at 30 s. The 13.0 wt% polypeptide membrane reached its maximal response with a 10% change in resistance. The 41.0 wt% polypeptide membrane produced a large response, reaching a maximal 30% change in resistance within 30 s. To account for the observation, we analyzed the surface morphology of the fabricated sensing material by using a scanning electron microscope (SEM). Figure 5 shows the SEM surface morphologies of (a) MWCNT; (b) MWCNT-polypeptide composite (13.0 wt%); (c) MWCNT-polypeptide composite (27.0 wt%); and (d) MWCNT-polypeptide composite (41.0 wt%) sensors. As shown in Figure 4, the top polypeptide layers of 13.0 wt% and 27.0 wt% could not cover the MWCNT completely. According to the SEM images, a higher percentage of polypeptide solution increased the MWCNT coverage proportion, and a 41.0 wt% concentration yielded the greatest coverage. This observation corresponded with the sensing experimental results shown in Figure 4. 

Consequently, because the 41.0 wt% polypeptide membranes produced the largest response among the three sample membranes, we used the 41.0 wt% polypeptide formulations to produce MWCNT-polypeptide membranes for the sensing experiments described as follows. 

### 3.3. Sensor Device Response

**Figure 6:**
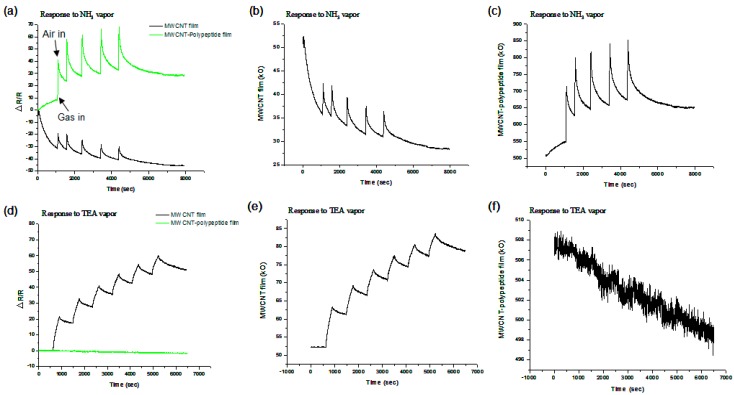
Gas sensing responses to NH_3_ gas (283 ppmv) of (**a**) MWCNT films and MWCNT-polypeptide composite films; (**b**) MWCNT films; and (**c**) MWCNT-polypeptide composite films, and gas sensing responses to triethylamine (2166 ppmv) of (**d**) MWCNT films and MWCNT-polypeptide composite films; (**e**) MWCNT films; and (**f**) MWCNT-polypeptide composite films.

The MWCNT-polypeptide composite films (41.0 wt%) were tested using two gases: ammonia and TEA. As illustrated in Figure 6a,b, upon exposure to the individual gases, the polypeptide-covered MWCNT conductive layer responded to amine gases were tested. As shown in Figure 6, the 40-mer polypeptide detected the presence of ammonia (283 ppm) and responded quickly upon exposure to the odor. However, although triethylamine was a strong-base VOC, no response from the sensing layer was observed, even with a higher concentration of TEA (2166 ppm) than that used in the ammonia test. Overall, the results of this study demonstrated the strong analytical potential of the polypeptide-based biosensor for detecting and differentiating specific types of VOCs.

### 3.4. Potential for Long-Term Sensing

**Figure 7:**
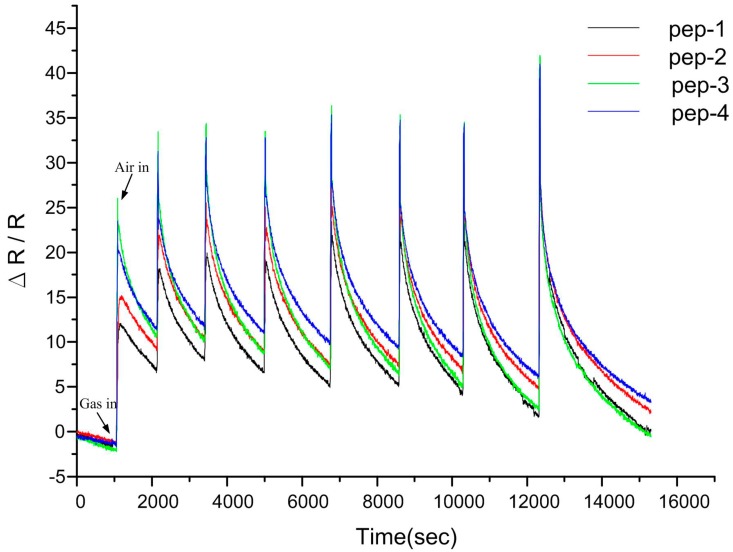
Long-term gas sensing responses to NH_3_ gas of four sensor devices fabricated with the same sensing materials (41 wt% MWCNT-polypeptide composite film).

Figure 7 presents the long-term gas sensing responses of the MWCNTs-polypeptide composite films to NH_3_ gas. The films were periodically exposed to NH_3_ gas to investigate their capacity for long-term sensing. The results revealed that the resistance changes reached at least 25%, even after eight exposures to NH_3_ and a testing time of nearly 10 h. The results further indicated that the peptide-based biosensor fabricated using our two-step approach could maintain stability and reproducibility, and thus, is a feasible candidate for onsite monitoring.

### 3.5. Discussion

The various types of functional groups of amino acid side chains are critical in determining the characteristics of a polypeptide. Our data revealed that the polypeptide-based sensing material in this study has considerable potential for recognizing polar gaseous chemicals according to size exclusion and interaction. Only smaller amine molecules appeared to reach and react with the sensing layer, leading to conductivity changes. Because of this encouraging observation, we propose testing various polypeptides of different lengths and compositions. Glycopeptides and even different lengths of sugar chains can also be used to construct a biosensor. By recording the resistance changes and establishing a databank by screening individual vapors, the polypeptide-based sensing films can be used in a wide variety of applications, such as to detect diseases or rotting fruit.

## 4. Conclusions 

We fabricated a conductive MWCNT-polypeptide two-layer composite material inspired by the structure of the olfactory system. Through a combination of conducting MWCNTs and a sensing polypeptide, this material provided enhanced sensitivity and is highly suitable for use as a microarray gas-sensing element. According to the sensing results, this two-layer polypeptide film exhibits higher sensitivity to small molecular vapor. We demonstrated that the polypeptide-coated sensor can detect ammonia instead of triethlyamine.
